# Joint EANM/SNMMI/ANZSNM practice guidelines/procedure standards on recommended use of [^18^F]FDG PET/CT imaging during immunomodulatory treatments in patients with solid tumors version 1.0

**DOI:** 10.1007/s00259-022-05780-2

**Published:** 2022-04-04

**Authors:** E. Lopci, R. J. Hicks, A. Dimitrakopoulou-Strauss, L. Dercle, A. Iravani, R. D. Seban, C. Sachpekidis, O. Humbert, O. Gheysens, A. W. J. M. Glaudemans, W. Weber, R. L. Wahl, A. M. Scott, N. Pandit-Taskar, N. Aide

**Affiliations:** 1 Nuclear Medicine Unit, IRCCS - Humanitas Research Hospital, Via Manzoni 56, 20089 Rozzano, Milano Italy; 2 The Department of Medicine, St Vincent’s Medical School, the University of Melbourne, Melbourne, Australia; 3 Clinical Cooperation Unit Nuclear Medicine, German Cancer Research Center (DKFZ), Im Neuenheimer Feld 280, 69210 Heidelberg, Germany; 4 Department of Radiology, New York Presbyterian, Columbia University Irving Medical Center, New York, NY USA; 5 Department of Molecular Imaging and Therapeutic Nuclear Medicine, Peter MacCallum Cancer Centre, Melbourne, Victoria Australia; 6 The Sir Peter MacCallum Department of Oncology, The University of Melbourne, Melbourne, Victoria Australia; 7 Mallinckrodt Institute of Radiology, Washington University School of Medicine, St. Louis, MO USA; 8 Department of Nuclear Medicine and Endocrine Oncology, Institut Curie, 92210 Saint-Cloud, France; 9 Laboratoire d′Imagerie Translationnelle en Oncologie, Inserm, Institut Curie, 91401 Orsay, France; 10 Department of Nuclear Medicine, Centre Antoine-Lacassagne, Université Côte d′Azur, Nice, France; 11 TIRO-UMR E 4320, Université Côte d′Azur, Nice, France; 12 Department of Nuclear Medicine, Cliniques Universitaires Saint-Luc, Université Catholique de Louvain (UCLouvain), Brussels, Belgium; 13 Nuclear Medical Imaging Center, Department of Nuclear Medicine and Molecular Imaging, University of Groningen, University Medical Center Groningen, Groningen, The Netherlands; 14 Department of Nuclear Medicine, Klinikum Rechts Der Isar, Technical University Munich, Ismaninger Str. 22, 81675 Munich, Germany; 15 Department of Molecular Imaging and Therapy, Austin Health, Studley Rd, Heidelberg, Victoria 3084 Australia; 16 Olivia Newton-John Cancer Research Institute, Heidelberg, Australia; 17 Faculty of Medicine, University of Melbourne, Melbourne, Australia; 18 School of Cancer Medicine, La Trobe University, Melbourne, Australia; 19 Nuclear Medicine Service, Department of Radiology, Memorial Sloan-Kettering Cancer Center, 1275 York Ave., New York, NY 10021 USA; 20 Nuclear Medicine Department, University Hospital, Caen, France; 21 INSERM ANTICIPE, Normandie University, Caen, France

**Keywords:** Positron emission tomography, PET/CT, [^18^F]FDG, guideline, immunotherapy, treatment response, malignant tumors

## Abstract

**Purpose:**

The goal of this guideline/procedure standard is to assist nuclear medicine physicians, other nuclear medicine professionals, oncologists or other medical specialists for recommended use of [^18^F]FDG PET/CT in oncological patients undergoing immunotherapy, with special focus on response assessment in solid tumors.

**Methods:**

In a cooperative effort between the EANM, the SNMMI and the ANZSNM, clinical indications, recommended imaging procedures and reporting standards have been agreed upon and summarized in this joint guideline/procedure standard.

**Conclusions:**

The field of immuno-oncology is rapidly evolving, and this guideline/procedure standard should not be seen as definitive, but rather as a guidance document standardizing the use and interpretation of [^18^F]FDG PET/CT during immunotherapy. Local variations to this guideline should be taken into consideration.

**Preamble:**

The European Association of Nuclear Medicine (EANM) is a professional non-profit medical association founded in 1985 to facilitate worldwide communication among individuals pursuing clinical and academic excellence in nuclear medicine. The Society of Nuclear Medicine and Molecular Imaging (SNMMI) is an international scientific and professional organization founded in 1954 to promote science, technology and practical application of nuclear medicine. The Australian and New Zealand Society of Nuclear Medicine (ANZSNM), founded in 1969, represents the major professional society fostering the technical and professional development of nuclear medicine practice across Australia and New Zealand. It promotes excellence in the nuclear medicine profession through education, research and a commitment to the highest professional standards. EANM, SNMMI and ANZSNM members are physicians, technologists, physicists and scientists specialized in the research and clinical practice of nuclear medicine. All three societies will periodically put forth new standards/guidelines for nuclear medicine practice to help advance the science of nuclear medicine and improve service to patients. Existing standards/guidelines will be reviewed for revision or renewal, as appropriate, on their fifth anniversary or sooner, if indicated. Each standard/guideline, representing a policy statement by the EANM/SNMMI/ANZSNM, has undergone a thorough consensus process, entailing extensive review. These societies recognize that the safe and effective use of diagnostic nuclear medicine imaging requires particular training and skills, as described in each document. These standards/guidelines are educational tools designed to assist practitioners in providing appropriate and effective nuclear medicine care for patients. These guidelines are consensus documents based on current knowledge. They are not intended to be inflexible rules or requirements of practice, nor should they be used to establish a legal standard of care. For these reasons and those set forth below, the EANM, SNMMI and ANZSNM caution against the use of these standards/guidelines in litigation in which the clinical decisions of a practitioner are called into question. The ultimate judgment regarding the propriety of any specific procedure or course of action must be made by medical professionals considering the unique circumstances of each case. Thus, there is no implication that an action differing from what is laid out in the guidelines/procedure standards, standing alone, is below standard of care. To the contrary, a conscientious practitioner may responsibly adopt a course of action different from that set forth in the standards/guidelines when, in the reasonable judgment of the practitioner, such course of action is indicated by the condition of the patient, limitations of available resources or advances in knowledge or technology subsequent to publication of the guidelines/procedure standards. The practice of medicine involves not only the science, but also the art of dealing with the prevention, diagnosis, alleviation and treatment of disease. The variety and complexity of human conditions make it impossible for general guidelines to consistently allow for an accurate diagnosis to be reached or a particular treatment response to be predicted. Therefore, it should be recognized that adherence to these standards/ guidelines will not ensure a successful outcome. All that should be expected is that practitioners follow a reasonable course of action, based on their level of training, current knowledge, clinical practice guidelines, available resources and the needs/context of the patient being treated. The sole purpose of these guidelines is to assist practitioners in achieving this objective. The present guideline/procedure standard was developed collaboratively by the EANM, the SNMMI and the ANZSNM, with the support of international experts in the field. They summarize also the views of the Oncology and Theranostics and the Inflammation and Infection Committees of the EANM, as well as the procedure standards committee of the SNMMI, and reflect recommendations for which the EANM and SNMMI cannot be held responsible. The recommendations should be taken into the context of good practice of nuclear medicine and do not substitute for national and international legal or regulatory provisions.

## Introduction

In the last decade, remarkable achievements in cancer treatment were made with the introduction of the immune checkpoint inhibitors (ICIs) [1, 2]. Most importantly, these have included the development and clinical introduction of antibodies against cytotoxic T-lymphocyte-associated protein 4 (CTLA-4) and programmed cell death-associated protein 1 (PD-1) and its ligand 1 (PD-L1). In 2011, ipilimumab, an anti-CTLA-4 monoclonal antibody, was the first to be approved by regulatory authorities based on the significant improvement in overall survival in melanoma [2]. Swiftly, this was followed by the development of monoclonal antibodies targeting PD-1, such as pembrolizumab and nivolumab, as well as those targeting PD-L1, such as avelumab and atezolizumab [3–6]. These agents act at differing points in T-cell activation with anti-CTLA-4 antibodies affecting early T cell priming and anti-PD-1 and PD-L1 affecting T-cell proliferation and cancer cell killing [7]. They can be used as single- or dual-agent therapies, or in combination with other standard oncological treatments including chemotherapy, radiotherapy or other targeted therapies [8–12]. The rapidly expanded clinical use of ICIs in the treatment of metastatic disease across a broad range of cancers was extended also to adjuvant and neoadjuvant settings in combination with curative intent surgery or radiotherapy of local or regional disease with a high risk of recurrence [12–14]. While durable responses can be achieved in a subset of metastatic patients, growing evidence suggests greater efficacy for these agents in the context of low tumor burden, even when conventional imaging does not depict measurable metastatic disease [15]. This is presumably achieved by targeting cancer cells before the development of an increasingly unfavorable tumor microenvironment, which, in turn, results in evolving resistance to immunotherapy treatments [16].

The immense progress of ICIs has brought challenges for cancer management, including a need for the oncological community to reconsider the conventional ways of assessing treatment efficacy and to develop strategies to manage a variety of relatively common immune-related adverse events (irAEs) that are not often encountered with other cancer therapies. It is now recognized that the effectiveness of ICIs is contingent on the successful activation of the multi-step cancer immunity cycle by the host immune cells. This begins with antigen presentation to dendritic cells and subsequently leads to priming, trafficking and infiltration of effector T cells into the tumor microenvironment [17]. Beyond conventional patterns of response as seen in chemotherapy, in a subset of patients unconventional modes of response were noted with delayed apparent efficacy of ICIs. These included a transient increase in tumor burden or even appearance of new lesions, termed “pseudoprogression,” which were attributed to initial recruitment of immune cells at tumor sites [18]. The standard radiographic criteria [19], commonly used to evaluate responses to chemotherapies or targeted therapies, did not account for these new kinetics of response. Hence, several modified evaluation criteria were devised to account for the appearance of new lesions and transient size increase by an extended delay to prove or refute tumor progression. These include immune-related response criteria (irRC) based on bidimensional measurements of target lesions or unidimensional size evaluation such as immune-related RECIST (irRECIST), immune RECIST (iRECIST) and immune-modified RECIST (imRECIST) [18, 20–22].

Cancer cells utilize aerobic glycolysis in part to fuel cell growth and energy needs [23], and this represents the fundamental pathway imaged by [^18^F]FDG PET/CT. Given the complexity of various immunotherapeutic responses, which challenged the conventional framework of RECIST, several studies evaluated the utility of [^18^F]FDG PET/CT in monitoring the response to ICIs [24–33]. As with morphological imaging, mechanistic differences in the mode of action of ICIs also challenged extrapolating the success of [^18^F]FDG PET/CT in monitoring cytotoxic or targeted treatment to evaluate immunotherapeutic strategies with checkpoint inhibitors [34]. Indeed, the very same metabolic reprogramming used by cancer cells extends to T cells, as both cancer cells and tumor infiltrating effector T cells possess high-affinity GLUT1 transporters to facilitate glycolysis [35]. This leads to a complicated interpretation of [^18^F]FDG PET/CT, especially early (in the first weeks/months) after treatment initiation, since increasing metabolic activity during therapy within the morphologically stable lesions paradoxically may indicate recruitment and activation of immune cells into the tumor microenvironment or draining lymphoid tissues rather than progression. Beyond response monitoring, [^18^F]FDG PET/CT has shown accuracy in monitoring systemic immune response and detecting irAEs in an early stage. PET/CT-derived quantitative metabolic parameters appeared to have prognostic implications [34, 36–39]. Therefore, there is an unprecedented need to provide imaging specialists and clinicians with a clinical practice framework for a more accurate, systematic and harmonized interpretation of [^18^F]FDG PET/CT in the rapidly evolving era of immunotherapeutic strategies.

## Goals

The goal of this guideline/procedure standard is to provide nuclear medicine professionals recommendations to correctly perform, interpret and report the results of [^18^F]FDG PET/CT in oncological patients undergoing immunotherapy, with a special focus on response assessment in solid tumors.

In addition, it provides general information to other professionals and medical specialties related to immune-oncology, i.e., radiologists, medical oncologists and radiation oncologists, for whom the acknowledgment of [^18^F]FDG PET/CT applications in this clinical context can be useful to support decisions regarding appropriate patient management.

This field is rapidly evolving, and this guideline/procedure standard cannot be seen as definitive, nor is it a summary of all existing protocols. Local variations to this guideline should be taken into consideration within a multidisciplinary setting.

## Definitions

Beyond the conventional imaging patterns of tumor response, such as complete/partial response and stable disease, ICIs have been associated with novel atypical patterns of response to treatment [40] that are not, or rarely, observed with conventional cytotoxic or targeted anticancer treatments.

### Pseudoprogressive disease

**Fig. 1:**
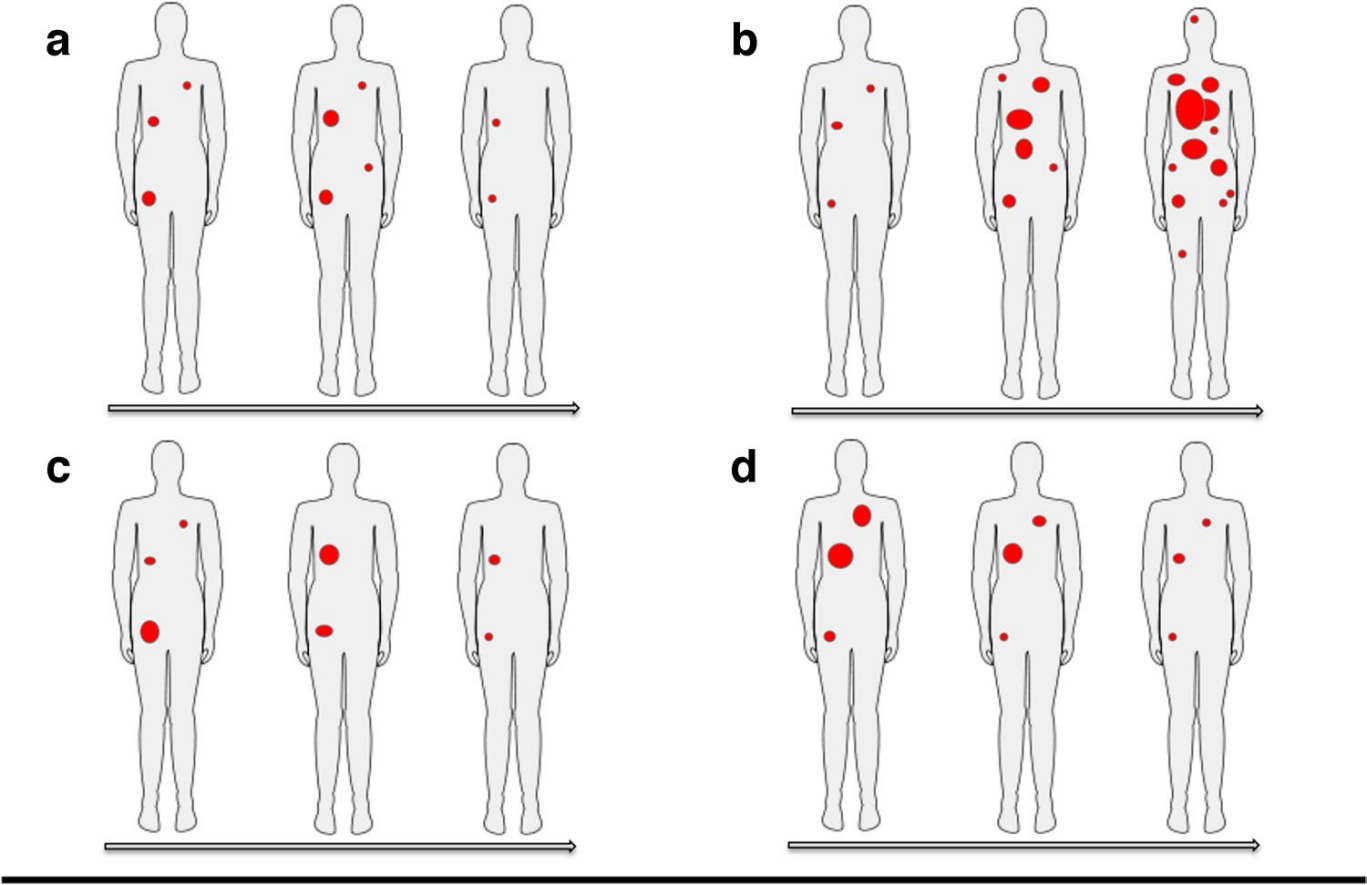
Illustration of four specific patterns of response to immunotherapy: a) pseudoprogression; b) hyperprogression; c) dissociated response; d) durable response

Historically, progressive disease is defined by an increase in size of target or nontarget lesions or by the appearance of new lesions (Fig. 1a). However, a series of clinical trials evaluating the efficacy of ipilimumab in melanoma demonstrated an unconventional pattern of tumor response on conventional imaging with transitory anatomical “progression” followed by response [41–43]. This pattern of response was named pseudoprogressive disease (PPD), and new response criteria were created [44, 45]. Most frequently, PPD occurs within the first 4–6 weeks of treatment, but can also occur up to several months after ICI initiation. The rate of PPD varies between tumor types and immunotherapies and has been reported in up to 10% of patients based on CT scan [46, 47] or [^18^F]FDG PET [48], appearing to be most common in patients with metastatic melanoma treated with anti-CTLA-4 antibody [46, 47], especially with combined ICIs. The PPD phenomenon has been attributed to a variety of mechanisms, including a delayed activation of immune response, local edema due to inflammatory processes and infiltration of immune cells within tumor lesions [49].

### Dissociated response

Dissociated response (DR), also known as mixed response or disproportional response (Fig. 1c), is defined by a decrease or stabilization in some tumor sites with a concomitant increase in other sites [50]. In patients treated with ICIs, DR has been reported in up to 10% of the cases [47, 48, 51]. A proportion of patients in retrospective cohorts benefit from prolonging ICI after a dissociated response, despite its classification as true progression by conventional response criteria. Some studies have demonstrated that a dissociated response is associated with a better prognosis than homogeneous progression of lesions in patients treated with ICI [48, 52]. This pattern of response may reflect the heterogeneity of tissue-specific tumor microenvironments, and can be easily detected by [^18^F]FDG PET due to its high sensitivity and capacity to assess early response on a lesion-by-lesion basis. From a clinical perspective, patients with dissociated response may benefit from treatment beyond progression potentially by continuing checkpoint inhibitor therapy and integrating local treatments, such as surgery, radiotherapy or interventional radiological treatment of oligoprogressive lesions [53].

### Hyperprogressive disease

Hyperprogressive disease (HPD) is defined as an atypical acceleration of tumor growth kinetics leading to premature death (Fig. 1b), which may occur following immunotherapy treatment [54–56]. Between 4% [57] and 29% [58] of patients with solid tumors will develop an augmented progression profile leading to a doubling of tumor burden and/or a twofold increase in tumor growth rate during ICI. There is currently no consensus on the precise criteria by which to define HPD, since previous studies used different methods of tumor burden assessment (for example, the sum of the largest diameters, tumor volume measurement) and different thresholds of tumor growth kinetics. In other cases, also a time to treatment failure under 2 months has been used to define HPD [57, 58]**.** Due to its recent emergence as a clinical phenomenon, HPD may be underdiagnosed. Therefore, the underlying mechanism of development represents an area of active investigation [59]. Some risk factors for HPD have been, however, described, and they include higher age [56] and the presence of MDM2/4 (murine double minute 2/4) family amplification or EGFR ( epidermal growth factor receptor) aberrations [57].

### Durable response

Depending on tumor and ICI type, 10 to 25% of patients with metastatic cancer will achieve a durable tumor response (Fig. 1d) that can be maintained several years even after stopping the treatment [60, 61]. A recent pooled analysis of phase III trials found that the proportion of patients who experienced a durable response was 2.3 times higher in those treated with an ICI compared with those treated by standard chemo/targeted therapies in the control arms (25% *vs* 11%) [62]. There is currently no standardized definition of durable response, since the criteria of durable response differ across studies.

## Clinical indications

The use of [^18^F]FDG PET/CT in the context of immunotherapeutic regimens should be considered at various time points related to treatment, based on clinical requirements. In particular:

- ***Before start of treatment***
  At baseline, [^18^F]FDG PET/CT should be considered mandatory for tumor assessment, for [^18^F]FDG-avid tumors, particularly in case of first-line immunotherapeutic regimens, since it provides a basis for tumor monitoring or a confirmation of disease progression/recurrence.
  Defining target lesions, the most intense sites of [^18^F]FDG uptake (e.g., SUVmax, SUVpeak) and computation of volumetric parameters (e.g., MTV) are recommended at baseline, to act as a basis for monitoring disease response at a later time.
- ***During the course of treatment***
  [^18^F]FDG PET/CT is recommended at interim, commonly 8-12 weeks (i.e., 3-4 cycles) after treatment start, in particular to complement the information obtained from morphological imaging with CT and to resolve discordant findings.
  The PET/CT scan can be also performed earlier or later during the course of treatment in case of clinical deterioration and/or suspicious progression on contrast-enhanced CT.
- ***Before immunotherapy discontinuation***
  In patients receiving maintenance therapy or undergoing long-term treatment with ICIs, [^18^F]FDG PET/CT may be obtained to assess metabolic response, particularly in partial responders or stable disease on CT [63].
  In patients requiring a temporary interruption of immunotherapy, [^18^F]FDG PET/CT restaging is recommended before restarting the treatment to reestablish a new baseline for subsequent response assessment.

## Response criteria

A wide range of response criteria to ICI (Table 1) have been proposed, typically relying on ceCT scans. Although these criteria account for pseudoprogression, they do not encompass the complexity of managing patients with ICIs due to several new patterns of response and progression described below. In addition, a wide range of treatment combinations is currently being explored. These extend from systemic ICIs monotherapy to combinations with chemotherapy or targeted therapies in cases with relevant tumor mutations (e.g., BRAF/MEK in melanoma and EGFR in non-small cell lung cancer, NSCLC). Other treatment variations include localized delivery of ICIs through intraarterial perfusion, local treatments with intratumoral immunotherapy [64] and radiotherapy–immunotherapy combinations [65, 66].

**Table 1:** Immune-related response criteria: [^18^F]FDG PET

Criteria	EORTC*	PERCIST 1.0*	PECRIT	PERCIMT	iPERCIST	imPERCIST5
Reference	Young et al. [67]	Wahl et al. [68]	Cho et al. [24]	Anwar et al. [26]	Goldfarb et al. [29]	Ito et al. [28]
Year	1999	2009	2017	2018	2019	2019
Tumor	Solid tumors	Solid tumors	Melanoma	Melanoma	NSCLC	Melanoma
Treatment	Chemotherapy	Chemotherapy, targeted therapies	Immune checkpoints inhibitors (anti-PD-1, anti-CTLA-4)	Immune checkpoints inhibitors (anti-CTLA-4)	Immune checkpoints inhibitors (anti-PD-1)	Immune checkpoints inhibitors (anti-PD-1)
Modality	FDG PET	FDG PET	CT and FDG PET	CT and FDG PET	FDG PET	FDG PET
Delay for confirmation of PMD	Undetermined	Undetermined	3–4 weeks	3 months	2 months	3 months
Target lesions	Tumor lesion with the highest SUV uptake	Minimum tumor SUL 1.5 × mean SUL liver, ≤ 5 target lesions/patient	RECIST 1.1 PERCIST 1.0	Size (metabolically active lesion) > 1.0 or 1.5 cm, ≤ 5 target lesions/patient	Minimum tumor SUL 1.5 × mean SUL liver, ≤ 5 target lesions/patient	Minimum tumor SUL 1.5 × mean SUL liver, ≤ 5 target lesions/patient
New lesions	Progression	Progression	Progression	Metabolic progressive disease (PMD)	Immune unconfirmed metabolic progressive disease (iuPMD)	Need to be included in the sum of SULpeak, PMD if > 30% increase in sum of SULpeak
Complete metabolic response (CMR)	Complete resolution of FDG uptake within the tumor volume so that it was indistinguishable from surrounding normal tissue	Disappearance of all metabolically active lesions	Disappearance of all lesions	Disappearance of all metabolically active lesions	Disappearance of any uptake in target lesion	Disappearance of all metabolically active lesions
Partial metabolic response (PMR)	A reduction of a minimum of 15–25% in tumor SUV after one cycle of chemotherapy, and > 25% after more than one treatment cycle	Reduction in sum of SULpeak in target lesions > 30% and absolute drop in SUL > 0.8 SUL units	≥ 30% decrease from baseline	Disappearance of some metabolically active lesions without any new lesion	Reduction in SULpeak in target lesions ≥ 30%	Reduction in sum of SULpeak in target lesions ≥ 30% and absolute drop in SUL by ≥ 0.8 SUL units
Stable metabolic disease (SMD)	An increase in SUV < 25% or a decrease < 15% and no visible increase in extent of FDG tumor uptake (> 20% in the longest dimension)	Neither PMD, PMR, nor CMR	Neither PD, PR or CR, evaluation of change in SUL peak of the hottest lesion: > 15.5% (clinical benefit), ≤ 15.5% (no clinical benefit)	Neither PMD, PMR, nor CMR	Neither PMD, PMR, nor CMR	Neither PMD, PMR, nor CMR
Progressive metabolic disease (PMD)	An increase in SUV > 25% within the tumor region defined on the baseline scan, visible increase in the extent FDG tumor uptake (> 20% in the longest dimension) or the appearance of new FDG uptake in metastatic lesions	Increase in sum of SULpeak of > 30% or the appearance of a new lesion	≥ 20% increase in the nadir of the sum of target lesions (> 5 mm)	≥ 4 new lesions of < 1 cm or ≥ 3 new lesions of > 1 cm or ≥ 2 new lesions of > 1,5 cm	≥ 30% increase in SULpeak or new metabolically active lesions: immune unconfirmed PMD (iuPMD)	> 30% increase in SULpeak, with > 0.8 SUL unit increase in tumor SULpeak
Confirmed PMD (cPMD)	na	na	na	na	PET at 4–8 weeks: confirmed PMD	na

* Although originally developed for chemotherapy and targeted treatments, EORCT and PERCIST criteria can be used even in case of immunotherapeutic regimens

At a clinical trial level, it has been demonstrated that reclassifying pseudoprogressive patients as treatment-sensitive avoids a potential bias that would otherwise favor chemotherapeutic alternatives based on progression-free survival (PFS), when, in fact, such patients may subsequently achieve prolonged survival. This phenomenon led to the refinement of standard response evaluation guidelines in the form of irRC [22], iRECIST [21] and irRECIST [69] for solid tumors. To prevent patients with PPD from prematurely terminating treatment, these guidelines propose a “wait-and-see” strategy (reevaluation using a follow-up scan 4–8 weeks later), when tumor burden appears to increase on imaging without significant clinical deterioration.

The interpretation of [^18^F]FDG PET/CT in patients treated with immunotherapy has to take into account this PPD phenomenon. Hence, several metabolic response criteria have been proposed as an alternative to PERCIST criteria [68]. These criteria are summarized below. Currently, there are insufficient data to decide which of these different approaches to categorize response is preferable. Furthermore, the impact on long-term patient outcomes has not been prospectively validated in randomized clinical trials.

In advanced melanoma treated with ICIs, PET/CT Criteria for Early Prediction of Response to Immune checkpoint inhibitor Therapy (PECRIT), which combines morphological (RECIST) and metabolic criteria, categorized 20 patients on the presence or absence of clinical benefit with a 100% sensitivity and a 93% specificity [24].

In an attempt to tackle the pitfalls and limitations of [^18^F]FDG PET imaging—foremost the phenomenon of pseudoprogression—in the assessment of immunotherapy response in melanoma, another set of modified response criteria has been developed, the PET Response Evaluation Criteria for IMmunoTherapy (PERCIMT). The cornerstone of PERCIMT is the finding that the changes in the absolute number of [^18^F]FDG-avid lesions are more predictive of clinical outcome than their respective standardized uptake value (SUV) changes during therapy with ICIs [26]. More specifically, according to these criteria, neither a mere increase in SUV of the target/index lesion(s) nor the development of one new hypermetabolic lesion in follow-up [^18^F]FDG PET/CT scan mean disease progression per se, as suggested by the conventional PERCIST/EORTC criteria [67, 68]. Instead, the application of a threshold of four newly emerged, [^18^F]FDG-avid lesions—with a decreasing cutoff of lesion number as the functional diameter of the lesions increases—can more correctly classify patients with progressive disease. More specifically, PMD is determined as the appearance of either four or more new lesions < 1 cm in functional diameter, or three or more new lesions > 1.0 cm in functional diameter, or two or more new lesions > 1.5 cm in functional diameter [26]. Otherwise, the patient can be classified as in PPD (Table 1).

PERCIMT criteria were developed in a metastatic melanoma cohort of 41 patients treated with ipilimumab, undergoing [^18^F]FDG PET/CT imaging before and after the end of ipilimumab treatment, and using the patients’ clinical response as reference [26]. They have been further validated in melanoma cohorts under different immunotherapeutic regimens and combinations both early during treatment (after two cycles of ICIs) [70, 71] and at the end of it (four cycles of ipilimumab and ipilimumab/vemurafenib treatment) [72, 73] yielding a satisfactory preliminary performance in patient stratification, predominantly in comparison with EORTC. However, further evaluation of the newly proposed criteria in larger patient cohorts is warranted, preferably in correlation with other metabolic or volumetric [^18^F]FDG parameters, such as MTV and total lesion glycolysis (TLG), as well as clinical and laboratory data.

Derived from PERCIST, imPERCIST differs in that the appearance of new lesions is not sufficient to classify a patient as having progressive disease. In these criteria, the peak standardized uptake value normalized for lean body mass (SULpeak) of up to five lesions on the baseline and follow-up scan is summed for each scan (maximum of 2 per organ). Target lesions on follow-up scans are the most intense lesions and are not necessarily the same as target lesions at baseline. PMD is defined as an increase of the sum of SULpeak by at least 30%. The appearance of new lesions is not sufficient to define PMD, but new lesions are included in the sum of SULpeak only if they show higher uptake than preexisting target lesions or if fewer than five target lesions were detected on the baseline scan. The prognostic value of imPERCIST criteria slightly outperformed those of standard PERCIST criteria [28].

Finally, a few recent studies have chosen to adapt the PERCIST criteria to the “wait-and-see” approach initially proposed by the iRECIST guidelines, leading to the so-called iPERCIST criteria [29, 48, 74]**.** Patients with new lesions or increase of more than 30% of the sum of SULpeak or of the SULpeak of the most intense lesions are classified as having unconfirmed progressive metabolic disease (uPMD). Then, if patients are clinically stable, reevaluation 4 to 8 weeks later is needed to establish confirmed progressive metabolic disease (cPMD). These studies have found that, for patients with metastatic lung cancer having uPMD on the first interim PET, the subsequent confirmatory PET reclassifies around one-third of these early-progressing patients as patients with atypical response patterns (PPD or dissociated response) who will in fact benefit from continuation of ICIs. Thus, it underlines the risk of falsely concluding to treatment failure after a first uPMD, a risk that is higher with [^18^F]FDG PET/CT than with ceCT due to its high sensitivity in detecting immune cell activation.

**Fig. 2:**
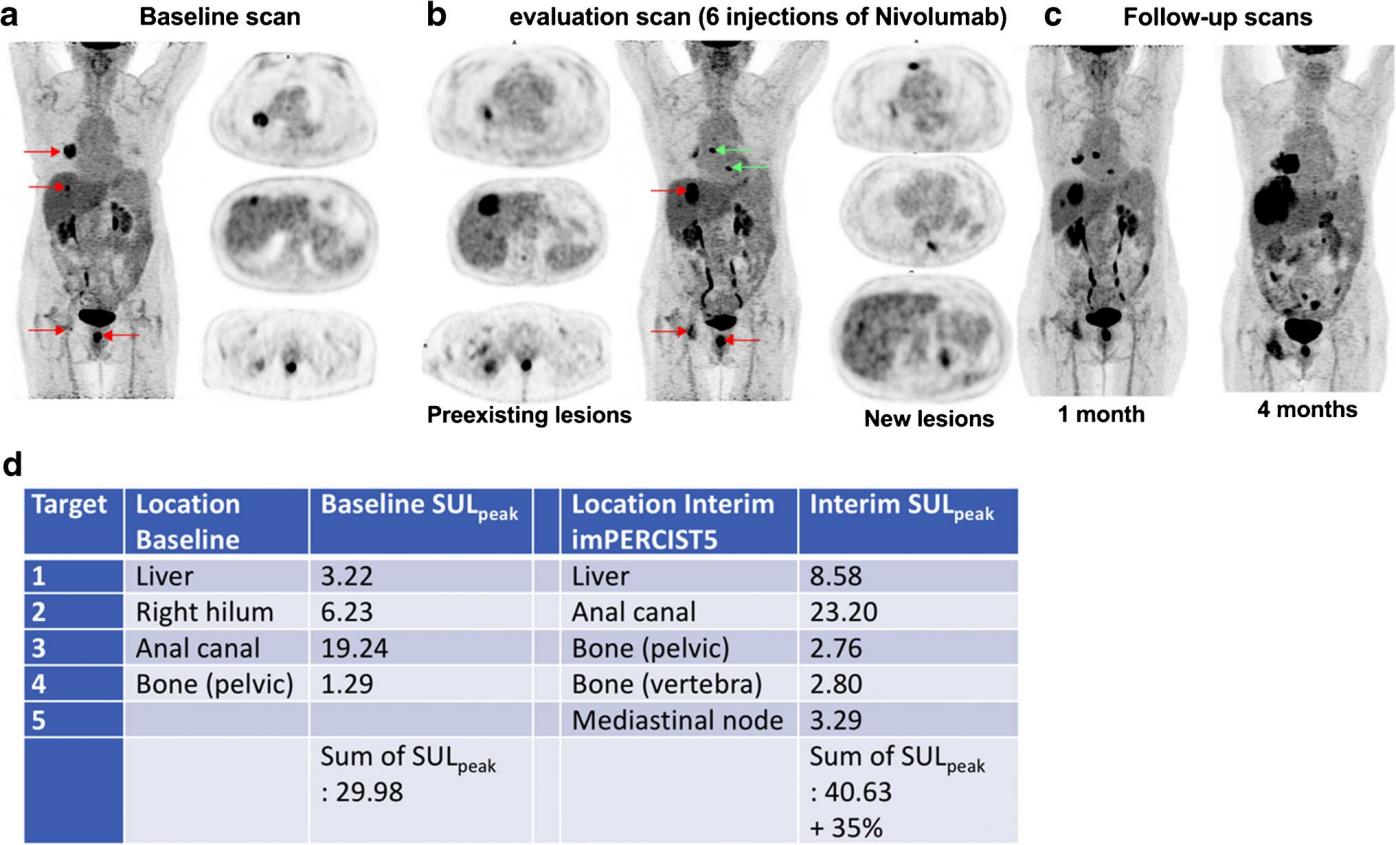
Illustration of target selection and [^18^F]FDG PET/CT response evaluation in a patient with multiple lesions and a dissociated response. Serial [18F]FDG MIP in a 73-year-old woman affected by a metastatic melanoma of the anal canal. (a) PET baseline before introduction of immunotherapy and (b) after six courses of nivolumab, showing a dissociated response with (i) progression of the main liver lesion, (ii) good response in the largest nodal lesion (right pulmonary hilum) and (iii) appearance of new lesions (liver, thoracic node, vertebral bone lesion; green arrows). This appearance of new lesions classifies the patient with progressive metabolic disease (PMD) according to the PERCIST criteria. As opposed to PERCIST, in imPERCIST5 (immunotherapy-modified PERCIST, five-lesion analysis), the appearance of new lesions alone does not result in PMD: PMD is defined only by an increase of the sum of SULpeaks by 30%, and new lesions are included in the sum of SULpeak if they show higher uptake than existing target lesions or if fewer than five target lesions were detected on the baseline scan. In the present case, a mediastinal node and a new bone lesion (green arrows) are selected, together with the three preexisting lesions (panel b). The patient is also classified as PMD according to imPERCIST. Follow-up scans at 1 and 4 months show clear progression (c). Summary table of target lesions, SULpeak values and their variation according to imPERCIST5 criteria are shown in panel (d)

**Fig. 3:**
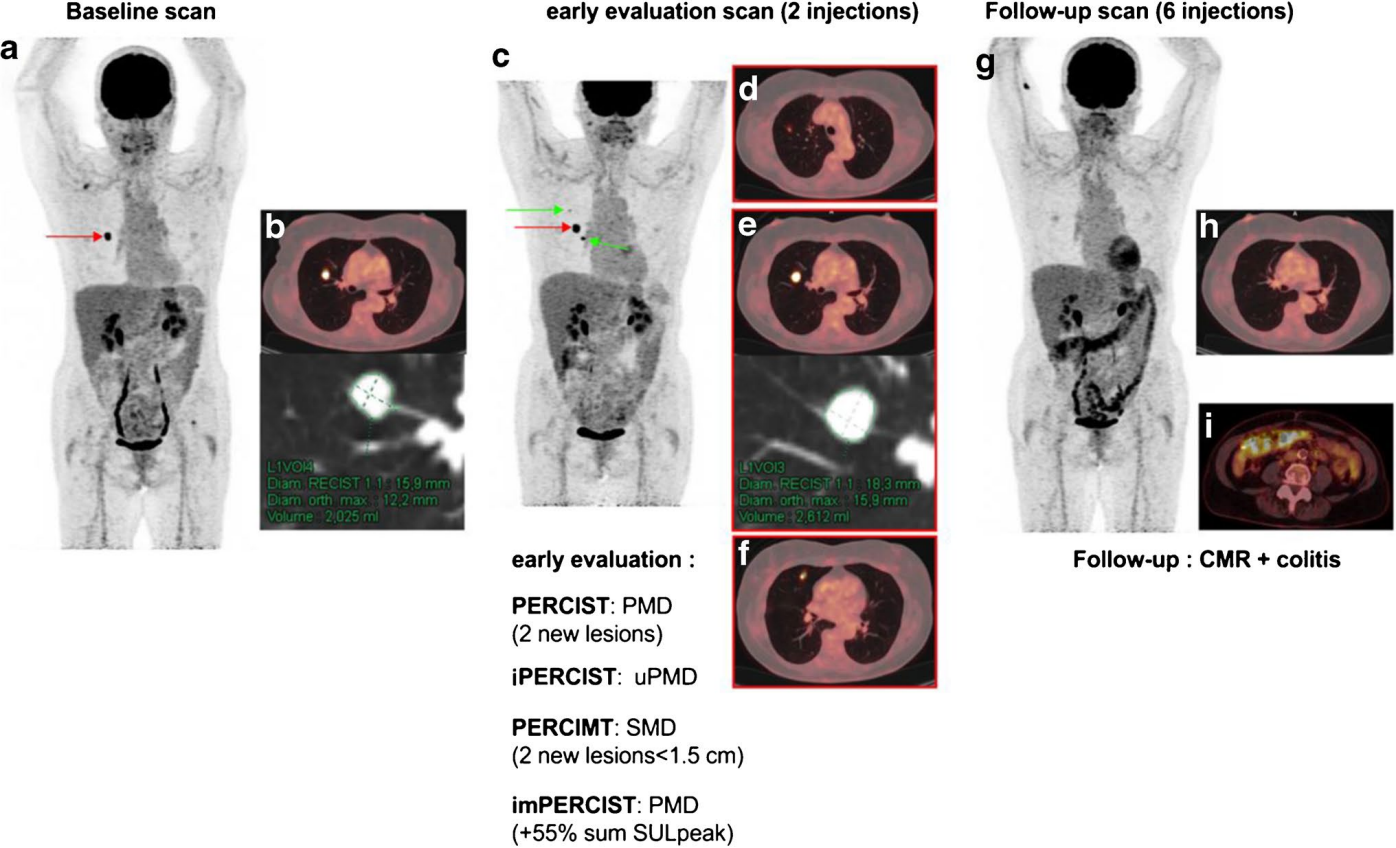
PERCIST, iPERCIST, imPERCIST and PERCIMT evaluation in a patient with pseudoprogression at early evaluation. Serial [18F]FDG MIP in a 66-year-old woman affected by a metastatic cutaneous melanoma. (a, b) MIP and transaxial slices at baseline before introduction of immunotherapy and after two courses of nivolumab (c-f), showing two new lung lesions (d, f; green arrows) as well as a progression in tracer uptake and RECIST measurements of the main lung metastasis (e; red arrows). This pattern classifies the patient with progressive metabolic disease (PMD) according to the PERCIST criteria, and uPMD based on iPERCIST criteria. imPERCIST including the two hottest lung lesions (e, f) also classifies the patient as PMD, due to an increase in the sum of SULpeak greater than 30%. According to PERCIMT, the patient is classified as SMD (appearance of two new lesions, the size of which is <1.5 cm). Follow-up scan shows complete disappearance of lung lesions, classifying the patient as CMR and retrospectively the early evaluation as pseudoprogression. Also noteworthy is the appearance of a diffuse colic uptake suggestive of colitis, confirmed also by wall thickening that is usually detected on CT images (g) and serves for the differential diagnosis between metformin-induced colon uptake from immune-related colitis [75]. This patient had a 23-month progression-free survival (PFS) and experienced a recurrence in the peritoneum and right adrenal gland with no active disease at the thoracic level

As general recommendation from this guideline/procedure standard, in case doubts exist between progression or pseudoprogression, especially on the first post-treatment evaluation, a confirmatory follow-up [^18^F]FDG PET/CT study 4–8 weeks later in the setting of clinical stability should be performed (Figs. 2 and 3). This consensus stems from the fact that there is no robust and externally validated tool to differentiate true progression from pseudoprogression based upon a single imaging assessment. Hence, treatment should be continued in clinically stable patients, absent excessive toxicity, to avoid discontinuation of ICIs in patients who may exhibit clinical benefit and objective response at a later time point.

## Assessing immune organs and irAEs

In addition to the [^18^F]FDG PET/CT response criteria cited above, that were created to meet the challenges raised by immunotherapy, several groups have reported baseline prognostic factors of response such as the MTV [76] and uptake in immune organs [77].

The first sign of immune activity to be evaluated is spleen enlargement and/or increased uptake leading to an equalization or an inversion of the spleen-to-liver ratio (SLR) [39, 78]. Some groups also proposed other signs such as the bone marrow-to-liver ratio (BLR) [79] and uptake in the ileocecal valve [80]. While the attention of the PET community has been mainly focused on the capability of SLR to predict immune activation [34, 78] (an increased spleen uptake being considered to reflect “unleashed” T lymphocytes with an expected better outcome), several studies (Table 2) showed that an increase in SLR on baseline or follow-up [^18^F]FDG PET was an unfavorable finding, likely related to inflammation and tumor burden.

**Table 2:** Summary of relevant signs of immune activation and their significance during ICIs

Study	Tumor type	ICI type	Number of patients	Metrics of immune activation	Conclusion
Wong et al. [39]	Melanoma	Ipi (50)	90	SLR	Baseline SLR > 1.1 is detrimental Only for Ipi
Prigent et al. [81]	Melanoma	Nivo (19) Pembro (9) Nivo + ipi (1)	29	SLR BLR	Increase > 25% of SLR_mean_ at 3 months is detrimental
Sachpekidis et al. [82]	Melanoma	Ipi	41	SUVmean, SUVmax, K1, k3, Ki, FD	Poor performance of spleen metabolism in predicting clinical benefit
Seban et al. [83]	Melanoma	Anti-PD-1	55	SLR BLR TMTV	TMTV* (> 25cm^3^), SLR (> 0.77) and BLR* (> 0.79) correlated with shorter survival
Seban et al. [84]	NSCLC	Nivo Pembro Atezo	80	TMTV	TMTV > 75 cm3 associated with shorter OS
Seban et al. [85]	Melanoma (Muc-M:24Cut-M:32)	Anti-PD-1 (45) Ipi (11)	56	TMTV BLR SLR	-Muc-M: increased SUVmax associated with shorter OS - Cut-M: increased TMTV and increased BLR independently associated with shorter OS, shorter PFS

*Remaining significant in a multivariable analysis

Strong reproducibility was reported for spleen and bone marrow measurements [81].

In addition to signs of immune activation and conventional or ICI-adapted [^18^F]FDG PET response criteria, the third cornerstone is evaluating the occurrence of irAEs. While several studies have shown that patients experiencing irAEs may have better survival [86, 87], studies on PET-detected irAEs are scarce [88, 89]. Lang et al. observed in a melanoma cohort under ipilimumab a significant correlation between PET signs of colitis and clinically significant diarrhea, although neither PET-colitis nor diarrhea was significantly correlated with response to therapy [90]. Recently, Wong et al. showed that [^18^F]FDG PET/CT could often detect relevant irAEs which may precede clinical diagnosis in melanoma patients receiving a combination of two ICIs (Table 3) [39, 91].

**Table 3:** Immune-related adverse events in patients with cancer treated with immune checkpoint inhibitors classified according to organ distribution and incidence [92–97]

Organ involved	Related irAEs	Overall incidence
Dermatological	Alopecia areata/universalis Dermatitis herpetiforme Erythema multiforme Granuloma annulare Lichen planopilaris/planus/lichenoid dermatitis Panniculitis/erythema nodosum Pemphigoid/pemphigus Psoriasis Pyoderma gangrenosum Sweet syndrome Vitiligo Stevens-Johnson syndrome Bullous pemphigoid DRESS symptoms Acute generalized exanthematous pustulosis Dermatomyositis	44–68% (anti-CTLA-4) 37–42% (anti-PD-1) 58–71% (combination therapy)
Endocrine	Adrenalitis Autoimmune diabetes mellitus Hyperparathyroidism/hypoparathyroidism (and hypocalcemia) Hypogonadism Hypophysitis Thyroiditis Insulitis (leading to IDDM)	1–3.9% (anti-CTLA-4) 0.5–10% (anti-PD-1) 7.7–20% (combination therapy)
Gastrointestinal	Enterocolitis Hepatitis Lymphocytic gastritis Pancreatitis	10–25% (anti-CTLA-4) 1–5% (anti-PD-1) 14–22% (combination therapy)
Pulmonary	Interstitial lung disease Pneumonitis Sarcoidosis Pleural effusions Reactive airway disease	3.8% (anti-PD-1) 9.6% (combination therapy)
Cardiac	Autoimmune myocarditis Myocardial fibrosis Autoimmune pericarditis Pericardial effusion Pericardial tamponade Cardiomyopathy with Takotsubo-like syndrome Acute heart failure Cardiac arrhythmia	0.5% (anti-PD-1) 2.4% (combination therapy)
Hematological	Thrombocytopenia Hemolytic Anemia Neutropenia Aplastic Anemia Pure Red Cell Aplasia Hemophagocytic Lymphohistiocytosis	0.5% (anti-CTLA-4) 4.1% (anti-PD-1) 4.7% (anti-PD-L1)
Musculo-skeletal	Myalgias/Myositis Dermatomyositis Arthralgia/polyarthralgia Arthritis/Enthesitis Fasciitis/eosinophilic fasciitis Jaccoud arthropathy Polymyalgia rheumatica Psoriatic arthritis Rheumatoid arthritis Spondyloarthropathy Tenosynovitis	1–23% (anti-CTLA-4) 2–26% (anti-PD-1 or anti-PD-L1) 9–43% (combination therapy)
Neurological	Aseptic meningitis Encephalitis Cranial nerve involvement Motor neuropathy Myasthenia gravis Neuromyelitis optica spectrum disorders Polyneuropathies Polyradiculopathies	3.8% (anti-CTLA-4) 6% (anti-PD-1) 12% (combination therapy)
Other	Systemic - Antiphospholipid syndrome - Lupus - Sarcoidosis - Sicca syndrome/Sjögren syndrome - Systemic sclerosis - Vasculitis Renal - Acute tubulointerstitial nephritis/renal tubular acidosis - Glomerulonephritis Ocular - Conjunctivitis - Episcleritis/scleritis - Orbital inflammation - Uveitis	0.6–5% (monotherapy) 10–12% (combination therapy)

## [^18^F]PET/CT protocols

### [^18^F]FDG PET/CT Acquisition

[^18^F]FDG PET/CT procedure should be performed as described in the EANM guideline [98] and SNMMI procedure standards for tumor imaging [99]. The RSNA QIBA FDG/CT guidance is largely concordant and also acceptable [100].

Briefly, fasting is recommended for at least 6 h, and the acquisition should be performed following an interval of 60 min from tracer injection (acceptable range of 55 – 75 min) [101] by using as default mode a torso imaging (from the skull base to the mid-thighs). Including the skull base at least for therapy assessment examinations is recommended, so that immune-related hypophysitis can be detected. An extended whole-body imaging, covering from the vertex through the feet, can be indicated in case of neoplasia with clinical suspicion of more extensive metastatic disease (e.g., NSCLC, melanoma, Merkel cell tumor, etc.). [^18^F]FDG PET/CT with diagnostic CT and/or contrast-enhancement can be used following the acquisition parameters determined according to specific radiological society guidelines [98, 102].

In case of repeated [^18^F]FDG PET/CT, especially in case of therapy response assessment, the scan should be performed with identical acquisition and reconstruction parameters, by maintaining stringent uptake intervals from tracer administration to image acquisition. In case of facilities with multiple tomographs, the repeated scan should be performed on the same machine. In any case, all scanners should comply with international harmonizing standards, such as the EANM/EARL program [98, 103].

### Data extraction and analysis

Quantitative PET/CT can be used as a diagnostic or prognostic tool (i.e., single measurement) or for therapy monitoring (i.e., longitudinal studies). Metrics include standardized uptake value (peak or max) computed either using bodyweight (SUV) or lean body mass (SUL) as normalizing measure for distribution volume, metabolic tumor volume (MTV) and total lesion glycolysis (TLG), defined as MTV × SUVmean. MTV is the volume inside a user- or algorithm-defined volume of interest (VOI) used to circumscribe the metabolically active tumor. Several techniques have been proposed to determine the limits of the VOI, threshold-based or algorithm-based [104], while TLG incorporates both [^18^F]FDG uptake and size of the tumor, also known as whole metabolic burden of the tumor [105]. The same method should be used to evaluate all scans—baseline and subsequent scans—in a patient as variability in MTV determinations by varying methods is well known.

Use of quantitative [^18^F]FDG PET/CT parameters for therapy monitoring purposes requires that these parameters are comparable among patients, regardless of the PET/CT system used. Therapy response criteria using SUVmax and, to a lesser extent, SUVpeak are affected by reconstruction inconsistencies between baseline and post-treatment scans [106, 107], which may arise when scanning patients in centers running several PET systems or as a result of patients’ mobility requiring scans at a different facility. Delineation of MTVs may be affected by the same errors as for SUVs, with variability in tumor delineation methodology being one of the major sources of variability [108]. It is therefore recommended to comply with harmonizing standards such as the EANM/EARL program, one of the international harmonization programs aiming at using [^18^F]FDG PET/CT as a quantitative imaging biomarker [98, 103].

## Documentation and reporting

General recommendations for [^18^F]FDG PET/CT documentation and reporting are available in the EANM guideline for tumor imaging [98] and SNMMI procedure standards [99]. Special considerations when dealing with ICIs are highlighted in the following.

- Clinical information
  The clinical history of the patient should be briefly summarized, including relevant diagnostic tests and prior imaging findings. The type and number of cycles of ICIs must be specified, including the date of the most recent administration. Concomitant drug and treatments potentially impacting [^18^F]FDG uptake should be listed. This particularly includes metformin to avoid misinterpreting increased colonic uptake as evidence of immunotherapy-related colitis [109]
- Technical Details
  Details of the administered activity, uptake interval, scanner used and blood glucose level should be recorded to allow these to be preferably emulated or, at least, compared for follow-up scans.
- Description of the findings
  Imaging findings should be described in a logical manner, preferably following relevance over clinical indication. Findings may be grouped by significance, TNM staging or described by body region. For important [^18^F]FDG findings, the location, extent and intensity of [^18^F]FDG uptake should be described, as well as the relevant morphological findings on CT.
  Target lesions should be identified following the indications of the chosen metabolic response criteria (Table 1) and reported, including longest diameter on axial view and SUV/SUL parameters (i.e., max, peak). In case of tumor response assessment, the pattern of metabolic response criteria used for the computation must be specified and documented.
  Comparison of current findings with prior scans and other comparative imaging, when available, must be performed and reported.
  The appearance, extent, severity and variation over time of the irAEs and other signs of immune activation (Tables 2, 3, and 4) must be described, preferably in a separate section of the report [34, 78, 110, 111].
- Impression/conclusions
  According to the clinical indication and timing, the conclusive remarks must be reported. Tumor extent or staging, treatment metabolic response and most relevant irAEs must be clearly stated. The complex nature of immune response seen with [^18^F]FDG PET/CT should be taken into consideration when performing conclusive remarks, with serial measurements and clinical response to be correlated. Ideally, there should be a focus on findings of clinical significance, particularly with respect to any additional diagnostic studies or subsequent scanning that might be required to clarify or confirm, for instance, the impression of disease progression (Table 4). Decisions based on the scan findings alone may be less accurate.
- Direct communication
  In addition to inclusion in the report conclusion, any significant abnormalities and severe irAEs [37, 39, 78] should be verbally communicated to the appropriate healthcare provider, in order to optimize patient management and avoid treatment delays that otherwise might result in significant morbidity or mortality. Reporting of abnormalities requiring urgent attention should be consistent with the policy of the interpreting physician’s local organization and the pathway for verbal communication.
- Additional remarks
  Taking into consideration that therapy assessment of cancer patients receiving ICIs (i) is often made in the context of busy PET centers and therefore should use user-friendly and reliable PET metrics, (ii) a consensus on treating patients beyond disease progression has been reached in certain tumor types and (iii) [^18^F]FDG PET/CT for assessment of immunotherapy is a dynamic field of research, these guidelines will provide recommendations on the use of the most clinically validated criteria, with metrics to be gathered for future pooled multicentric research. Participation of PET readers in tumor boards is crucial for patient care. Furthermore, [^18^F]FDG PET should be included in prospective randomized clinical trials in order to determine whether [^18^F]FDG PET-based response assessment can predict the effectiveness of a specific drug regimen more accurately than response assessment by RECIST. It should be recognized that the use of PET may alter the rate of detecting irAEs compared to that previously identified in routine clinical care and may impact both subsequent treatments that might be administered for these complications and the longer-term consequences.
  The implementation of artificial intelligence (AI) for image analysis with the development of dedicated algorithms for PET image segmentation as well as AI-based evaluation of follow-up studies will facilitate therapy monitoring with [^18^F]FDG PET–CT in future [112–114]. Additionally, the translation of dedicated tracers into clinical routine, like labeled T cells, or labeled PD-1 or PD-L1 antibodies will provide complementary information to [^18^F]FDG and improve prediction to immunotherapy response [115].

**Table 4:** Checklist of requirements and recommendations to consider when approaching [^18^F]FDG PET/CT imaging during treatment with ICIs

	CHECKLIST
HISTORY	Type of immunomodulatory treatment • ICI: e.g., anti-CTLA-4, anti-PD-1/PD-L1 or combination • Intratumoral immunotherapies • Cell-based immunotherapies Number of cycles received and date of the last injection If intratumoral administration the site of injection Prior lines of treatment If combination treatment, the type of treatment or, in case of radiation, the site of irradiation Clinical symptoms suggesting immune-related adverse events For diabetic patients, check whether drugs likely to mimic colitis (biguanides) have been given Previous or ongoing use of corticosteroids and antibiotics should be noted
THERAPY RESPONSE	Response of target lesion(s) If the appearance of new lesions: • The number of new anatomical sites and the number of new lesions • Could new lesions be explained by other processes such as sarcoidosis or other irAES? • If new nodal sites ○ located in the drainage area of the main tumor? ○ In a distribution suggestive of sarcoid-like lymphadenopathy If possible, include MTV/TLG at baseline and subsequent studies If there is doubt whether there is progression or pseudoprogression, especially on the first post-treatment evaluation and a confirmatory follow-up [^18^F]FDG PET/CT study in > 4 weeks later in the setting of clinical stability or biopsy should be recommended
ASSESSING IMMUNE ORGANS	Report spleen-to-liver ratio
MANIFESTATIONS OF IMMUNE-RELATED ADVERSE EVENTS	Different ICIs have differing side-effect profiles (e.g., colitis is more common in anti-CTLA-4 and pneumonitis more common in anti-PD-1/PD-L1) Sarcoidosis can have variable presentations and can involve lymph nodes and other organs Increased bone marrow activity and inversion of spleen-to-liver [^18^F]FDG uptake ratio may support systemic immune response If available comparison with baseline study, it is critical to monitor the metabolic activity within the organs Preferably, the brain should be included, [^18^F]FDG uptake in the pituitary gland should be checked and compared it to the baseline study When immune-related adverse events are shown on [^18^F]FDG PET/CT check patient’s recovery on subsequent studies

## Checklist

Table 4 highlights the checklist of requirements for patients with solid tumors treated with ICI.

## Liability statement

This guideline summarizes the views of the EANM Oncology and Theranostics Committee, the EANM Inflammation and Infection Committee, the SNMMI and the ANZSNM. It reflects recommendations for which the EANM cannot be held responsible. The recommendations should be taken into context of good practice of nuclear medicine and do not substitute for national and international legal or regulatory provisions.

## Abbreviations

AI: Artificial Intelligence
ANZSNM: Australian and New Zealand Society of Nuclear Medicine
BLR: Bone marrow-to-liver ratio
BRAF: B Rapidly Accelerated Fibrosarcoma
CMR: Complete metabolic response
cPMD: Confirmed progressive metabolic disease
CR: Complete response (anatomical)
ceCT: Contrast-enhanced CT
CT: Computed tomography
CTLA-4: Cytotoxic T-lymphocyte-associated protein 4
DR: Dissociated response
EANM: European Association of Nuclear Medicine
EARL: EANM Research Ltd
EGFR: Epidermal growth factor receptor
EORTC: European Organization for Research and Treatment of Cancer
FD: Fractal dimension
FDG: 2-[^18^F]fluoro-2-deoxy-D-glucose
HPD: Hyperprogressive disease
ICIs: Immune checkpoint inhibitors
IDDM: Insulin-Dependent Diabetes Mellitus
imPERCIST: Immunotherapy-modified PERCIST
irAEs: Immune-related adverse events
iRECIST: Modified RECIST 1.1 for immune-based therapeutics
irRECIST: Immune-related Response Evaluation Criteria in Solid Tumors
MDM 2/4: Murine double minute 2/4
MEK: Mitogen-activated protein kinase
MIP: Maximal intensity projection
MTV: Metabolic tumor volume
NSCLC: Non-small cell lung cancer
PECRIT: PET/CT Criteria for Early Prediction of Response to Immune checkpoint inhibitor Therapy
PERCIMT: PET Response Evaluation Criteria for IMmunoTherapy
PERCIST: Positron Emission Tomography Response Criteria In Solid Tumors
PD: Progressive disease
PD-1: Programmed cell death-associated protein 1
PD-L1: Programmed death ligand 1
PET/CT: Positron emission tomography/computed tomography
PFS: Progression-free survival
PMD: Progressive metabolic disease
PMR: Partial metabolic response
PPD: Pseudoprogressive disease
PR: Partial response
QIBA: Quantitative Imaging Biomarkers Alliance
RECIST: Response Evaluation Criteria in Solid Tumors
RSNA: Radiological Society of North America
SD: Stable disease
SLR: Spleen-to-liver ratio
SMD: Stable metabolic disease
SNMMI: Society of Nuclear Medicine and Molecular Imaging
SUL: Standardized uptake value normalized by lean body mass
SUV: Standardized uptake value
TLG: Total lesion glycolysis
TMTV: Total metabolic tumor volume
TNM: Tumor (T), nodes (N), and metastases (M)
uPMD: Unconfirmed progressive metabolic disease
VOI: Volume of interest
